# Structural Solutions for Low-Cost Bamboo Frames: Experimental Tests and Constructive Assessments

**DOI:** 10.3390/ma9050346

**Published:** 2016-05-07

**Authors:** Mauro Sassu, Anna De Falco, Linda Giresini, Mario Lucio Puppio

**Affiliations:** Department of Energy, Systems, Territory and Constructions Engineering, University of Pisa, Largo Lazzarino 1, Pisa 56122, Italy; a.defalco@ing.unipi.it (A.D.F.); linda.giresini@unipi.it (L.G.); mariolucio.puppio@ing.unipi.it (M.L.P.)

**Keywords:** bamboo, low-cost construction, full scale testing, experimental tests, one-storey buildings

## Abstract

Experimental tests and constructive assessments are presented for a simple bamboo framed structure with innovative low-cost and low technology joints, specifically conceived for small buildings in developing countries. Two full scale one-storey bamboo frames have been designed by using the simplest joints solution among three different tested typologies. The entire building process is based on low-technology and natural materials: bamboo canes, wooden cylinders, plywood plates and canapé rods. The first full scale specimen (Unit A) is a one-storey single deck truss structure subjected to monotonic collapse test; the second full scale specimen (Unit B) is a one-storey double deck truss structure used to evaluate the construction time throughout assembling tests. The first full scale specimen showed ductility in collapse and ease in strengthening; the second one showed remarkable ease and speed in assembling structural elements. Finally several constructive solutions are suggested for the design of simple one-storey buildings; they are addressed to four purposes (housing, school, chapel, health center) by the composition of the proposed full scale bamboo frames. Ease of use and maintenance with a low level of technology contribute to application in developing countries although not exclusively.

## 1. Introduction

Bamboo is a low-cost building material, widely available in the world; it is lightweight, durable, flexible, and easily cultivated and processed. The use of bamboo as a structural material is well-known in constructive traditions and in recent innovations. Its mechanical properties have been studied from several points of view: e.g., in [1,2] the micromechanical role of fiber and matrix of bamboo as a “natural composite material” in [3,4] different typologies of bamboo plants and in [5,6] the non-linear constitutive behavior of bamboo. Moreover, the capability of protecting the environment, due to its sustainability, is a relevant aspect to consider, as discussed in [7,8,9], and its ability to mitigate constructive emergencies in developing countries [10,11].

Examples of bamboo usage, as a composite material joined with other structures or organized throughout fibers, demonstrate its full potential as a structural and environmental friendly material. The research into the reduction of environmental impact and/or the use of cheap or recycled materials [12] represent viable ways to find low-cost structural solutions. Natural reinforcing materials were coupled with concrete to analyze the increase in strength and sustainability [13,14,15]. New biocomposite materials with bamboo strips [16] and composed bamboo elements [17] were proposed to extend the traditional use in scaffolding [18] to structural applications. The use of bamboo as a natural construction material is also regulated in several countries by specific standards [19,20,21,22,23]. Great relevance also emerged in the field of seismic performance of civil constructions [24,25] where structural joints play a fundamental role. The topic was in depth discussed particularly for developing countries [26,27,28,29]. A frequent field of application for bamboo is the construction of low cost light roofs for one-storey buildings: the reduction of masses at the top of the building implies minor static and seismic actions.

The reduced ductility of bamboo elements is a critical aspect from structural point of view: bamboo is characterized by brittle behavior, mainly for tension forces, and the collapse is, in most cases, caused by longitudinal cracks along the weakest lines. Another negative aspect for traditional bamboo frames is the relative weakness of the joint between the canes. Often the nodes are built, in vernacular contexts, throughout tied ropes or by incisions of the canes, simple to realize but not suitable to transmit the entire bearing capacity of the bamboo elements. By contrast, modern technologies allow joints with high structural features, but complicated technologies or materials are not easy to supply or disseminate in developing countries [30,31,32,33]. The optimization of the mentioned aspects is here investigated, starting from previous activities performed by the authors. Three low-cost solutions for bamboo joints are here summarized to harmonize structural efficiency with constructive simplicity: joints with (i) steel nails, bolts and steel plates [34,35]; (ii) steel nails and bolts with plywood plates [36,37]; and (iii) wooden bolts, canapé rods and plywood plates [38,39]. Experimental tests on each joint solution have been performed and compared to verify economy and easiness in assemblage with static and seismic performances. The third joint type, corresponding to the lowest cost and technology requirements presents the highest environmental impact and ease of assembly. That solution is adopted to execute two full scale trussed specimens also illustrated in the paper.

The first full scale specimen (Unit A) tested in the present paper is a single deck trussed structure, made with a couple of frames with a wooden roof, loaded up to collapse with monotonic vertical loads. The second full scale specimen (Unit B) is a symmetrical double deck frame, with Polanceau beam and coupled columns, subjected to assembling tests. The collapse test showed the global ductility behavior of the bamboo frame in the presence of vertical loads. The failure mechanism, involving only wooden pins in a joint with large inelastic deformations, prevented brittle collapse of the other elements such as bamboo canes. It therefore determines a safe structural behavior avoiding any brittle collapse and also permitted an economical repair with the simple substitution of the pins. Analytical evaluations confirm the ductile behavior attained by the third joint solution, by comparing expected performances with experimental results. In addition, the assemblage tests, held by a group of un-skilled workers, showed the ease and the speed of the constructive phases.

Finally several buildings plans with Units A and B in modular schemes are presented for typical one-storey buildings such as housing, schools, small churches and health centers; the aim of this is to contribute to proposals for self-construction activities and encourage their dissemination among building workers in developing countries.

## 2. Experimental Tests on Joints

In this section a series of static tensile tests on three types of joints for bamboo structures are discussed. From a structural point of view, the main collapse mechanisms that can be expected during the tensile tests have been the following:

1. shear-bending failure of wooden pins (weak pins);
2. hole deformation in plywood plate (weak plywood plate);
3. tensile tearing-out of bamboo canes (weak bamboo canes by axial force);
4. longitudinal cracks in bamboo canes between the holes (weak bamboo canes by shear).

### 2.1. Joint with Screw Nails and Steel Plate

**Figure 1:**
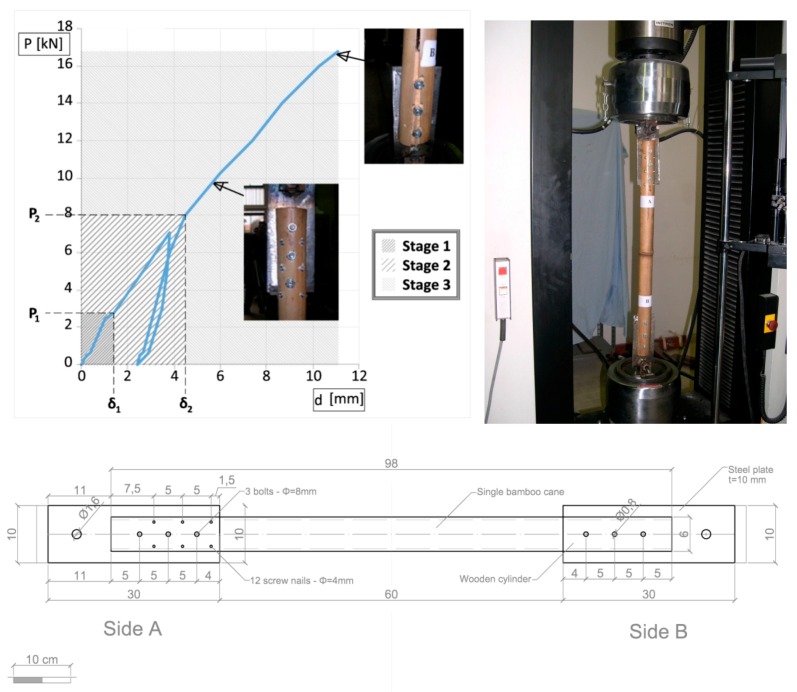
Tensile test on the 1st joint type.

The first typology of joint has been tested [34,35] on a single bamboo cane (Bambusa Vulgaris, diameter D = 60 mm, thickness *s* = 5 mm). The joint is reinforced by means of a wooden cylinder and connected to 10 mm thick metal plate (Figure 1). The lower extremity of each rod (joint B) is connected by three bolts (D = 8 mm) and resin glue and the upper extremity (joint A) by three bolts (D = 8 mm) and twelve screw nails (D = 4 mm). The tensile monotonic tests are conducted by means of an INSTRON Mod1186 at the Structural Laboratory of the University of Pisa, with a force full scale of 1000 daN. The relative displacement of upper and lower joint was monitored with four LVDT transducers (precision 1/100 mm). The four specimens showed an average axial force-displacement diagram (Figure 1) with three mechanical phases (elastic, inelastic and collapse) as described in [34,38]. The lesson, learned by the tests on the first type of joint, is that it can transfer a high percentage of the bamboo strength but does not present a significant ductile phase. Furthermore several of the used materials are high technology and energy consuming (steel plates, bolts, screw nails, glue).

### 2.2. Joint with Screw Nails and Plywood Plate

**Figure 2:**
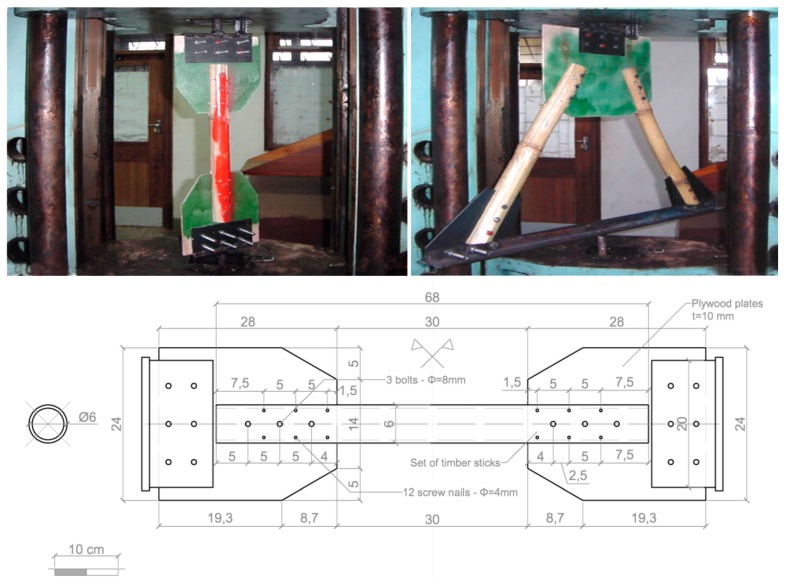
Tensile tests on single and double 2nd type joints.

The second joint proposal is meant to produce a simpler solution and higher ductility level compared to the first one. Plywood plates are used in place of steel plates and the internal part of bamboo cane has been filled with a set of wooden sticks instead of a unique wooden cylinder. An experimental campaign was carried out at the Polytechnic of Malawi Laboratory [36,37] with an Avery-Denison at a resolution of 50 daN and a maximum load of 1000 kN, arranging a set of nodes with single or coupled connection (Figure 2). The first consists in a single rod tensile test, with specimen formed by two plywood plates bolted at the extremities, connected to the tension machine through a couple of steel supports (these supports were not considered testing joints). The second regarded a couple of bamboo canes through plywood plate and steel nails designed with a vertical angle of about 60°. Also these specimens were linked to the tension machine through steel elements. The bamboo rods and the plywood plate were mutually joined by three steel bolts of 8 mm diameter; a set of timber sticks were inserted at the extremity of each rod to strengthen the connection. With the purpose of improving the connection of the bamboo rod with the plywood plate and the timber sticks, six screw nails of diameter 4 mm and length of 40 mm were also added to the bamboo joints. The lesson learned from the tests on the second type of joint is that the ductile behavior was activated through the deformation of the plywood around the holes; regardless, a subset of high technology elements (bolts, screw nails, glue) were not removed.

### 2.3. Joint with Wooden Pins and Plywood Plates

**Figure 3:**
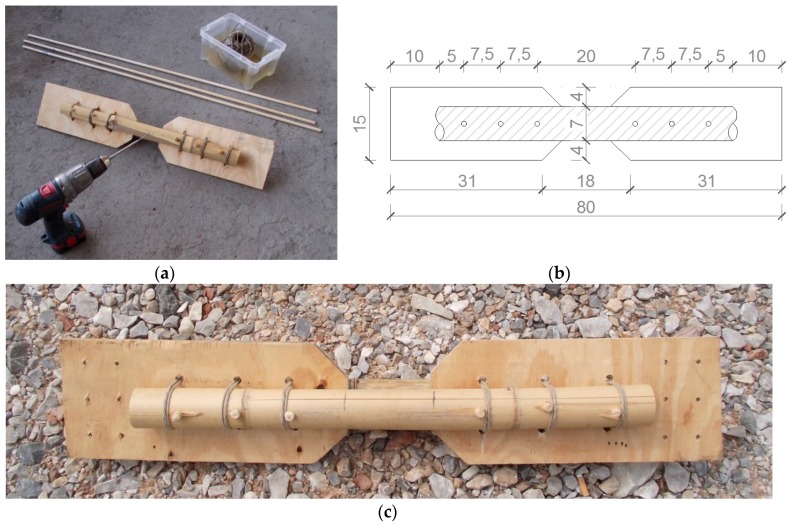
3rd type of joint with drill and materials used (**a**,**c**) and dimensions in cm (**b**).

The third solution is oriented to a further simplification with respect to the previous proposals. The joint is now formed by a couple of bamboo rods connected by wooden pins, instead of steel nails, without any insertion of glue and wooden reinforcements within the bamboo elements; the joint is also transversely strengthened by canapé ropes [38]. The entire constructive phases are conducted only with a portable drill, to put holes in bamboo rods and plywood plates, a hammer to put wooden pins into the holes, and a saw to cut the wooden pins. All the instruments are simple to supply and to use, while all the structural materials (bamboo, plywood, wooden cylinders, canapé ropes) are low-technology and easy to supply (Figure 3).

**Figure 4:**
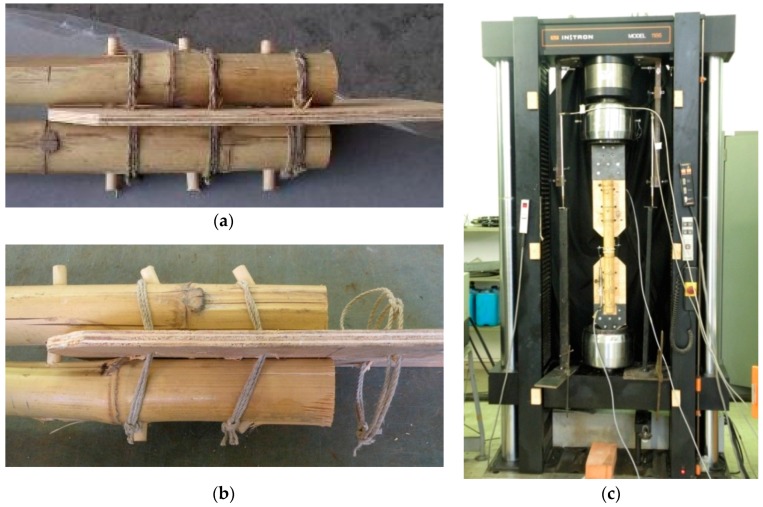
3rd type of joint before (**a**) and after (**b**) collapse and testing machine (**c**).

**Figure 5:**
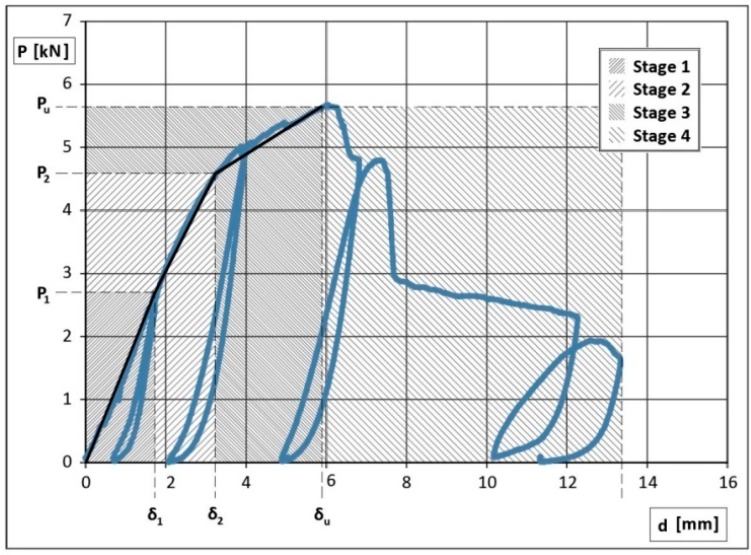
Force P (kN)–displacement d (mm) diagram for the 3rd joint type.

Tension tests have been performed on the specimen (Figure 4) composed of two bamboo rods (Bambusa vulgaris, from Southern China) with a diameter of 50 mm and thickness of 5 mm, a plywood plate with a thickness of 8 mm, a canapé rope with a diameter of 3 mm. A couple of steel plates, designed to avoid premature collapses on the gripping area, are able to connect the specimen to the tension machine. The canapé wires have been manually forced around the bamboo elements through holes in the plywood. The tests were executed on three specimen pairs on the same machine of the 1st type of joint, controlling the relative displacement of upper and lower joint with four LVDT transducers (precision 1/100 mm). Bamboo was qualified with compression and shear tests as in ISO 22157-1 and ISO 22157-2 [23]. The cyclic tension tests, up to collapse, were carried out with increasing load cycles. The force-displacement diagram from experimental results is reported in Figure 5 [38,39].

The mechanical behavior of the connection can therefore be expressed in four phases:

1. the specimens show elastic deformations without relative displacements between plywood and bamboo (elastic phase);
2. the wooden pins are deformed by bending moment inducing relative displacements between plywood and bamboo (slipping phase);
3. the wooden pins are affected by inelastic large deformations with consequent wide relative displacements up to maximum force (plastic phase); softening stress-strain curve is caused by the progressive failure of the wooden pins (starting from the extremity pins), partially constrained by the canapé wires (softening phase).

## 3. Structural Design of the 3rd Type of Joint

The design of the 3rd type of joints is meant to favor the first mechanism (“weak pins”, see Section 2), displayed in Figure 4b. In fact the collapse of wooden pins of diameter 9 mm protects bamboo elements from brittle damage. Moreover the collapse of wooden pins impedes also inelastic deformations of the plywood plates; this implies a simple repair operation, by replacing the wooden bars and the canapé rods without other interventions such as more complicated substitution of bamboo rods or plywood plates.

The observed ductility factor of node *μ* = *d_u_*/*d*_1_ during experimental test is between 3.2 and 4.6; it therefore shows a satisfactory ductile behavior, in spite of the bamboo brittleness, without significantly reduced collapse force. In fact the classic formula of the design tensile load of bamboo cane (ISO/DIS22156) with net area *A* and ultimate stress *f_k_* is:

(1) $$P_{d,b}=\;\frac{A\;f_{k}\;G\;D}{\gamma_{m}}$$

where *G* is a load factor between 1.0 and 1.5, *D* is a reliability factor in execution (mainly 0.5) and *γ_m_* the bamboo safety factor (2.25). In the considered example, given *A* equal to 13.7 cm^2^ and *f_k_* of 18 MPa, the maximum tensile load $P_{d,b}$ is between 5.50 and 8.25 kN which is higher than the wooden pins strength. From another hand, the conventional design formula for the resisting force $P_{d,p}$ of the weak pins mechanism could be the following:

(2) $$P_{d,p}=\;\tau_{p}n_{p}n_{s}\;\pi \frac{d^{2}}{4}$$

where $n_{p}$ is the number of wooden cylinders, d the diameter, $n_{s}$ the active shear sections and $\tau_{p}$ the equivalent shear stress. From Equation (2), an elastic limit of shear stress can be obtained for the pins, by imposing $P_{d,p}=P_{1}$, where $P_{1}$ is the elastic limit value shown in Figure 5:

(3) $$\tau_{p,e}=\;\frac{4\;P_{1}}{n_{p}n_{s}\;\pi \;d^{2}}$$

By substituting $P_{1}=2.85$ kN and assuming $n_{p}=3$, $n_{s}=2$, d=9 mm, the average value of the elastic limit shear stress for pins is $\tau_{p,e}=7.5\text{ MPa}$. Analogously, the corresponding $P_{2}$ value—equal to 4.65 kN—provides an ultimate limit shear stress $\tau_{p,u}=\;12.2\text{ MPa}$. It is important to point out that, with respect to the 2nd type of joint, the decrement of the ductility is low, while a relevant increment of the collapse displacement is detected. This is due to the wide softening phase caused by the role of canapé rods, as shown in Figure 4 and Figure 5.

Considering the average value of the ultimate displacement *d_u_* = 7.97 mm, it would be possible to deduce an estimation of the work done during the cyclic deformations, by evaluating the area under the diagram of Figure 5.

Another aspect related to the 3rd type of joint consists in the fact that the single rod test is representative even of the mechanical behavior of multiple rod joints. It is important to emphasize that the collapse of wooden pins is independent from the number of rods converging on the node.

Moreover, the entire constructive system is able to favor a precast activity. A unique shape of plywood plates can be used for several spans of frames, due to the homotheticity of the truss nodes. Last but not least, the entire set being eco-friendly materials is a remarkable value in favor of this last type of joint.

## 4. Experimental Tests on Full Scale Models

### 4.1. The 1st Full Scale Model (Unit A)–Collapse Test

**Figure 6:**
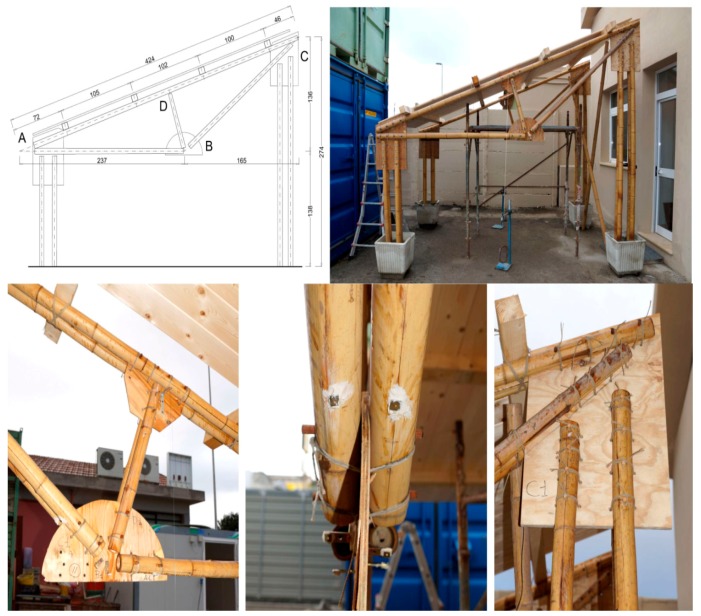
The 1st full scale specimen (Unit A) and the assembling phases.

The full scale specimen named unit A (Figure 6) is formed by a couple of frames, with a span of about 375 cm, at a distance of about 200 cm from each other; the roof is a wooden plate of 30 mm supported by four purlins. The adopted joints are of the 3rd type (Section 2.3 and Section 3) while the columns, composed by two couple of bamboo canes, are fixed in pots filled with sand.

**Figure 7:**
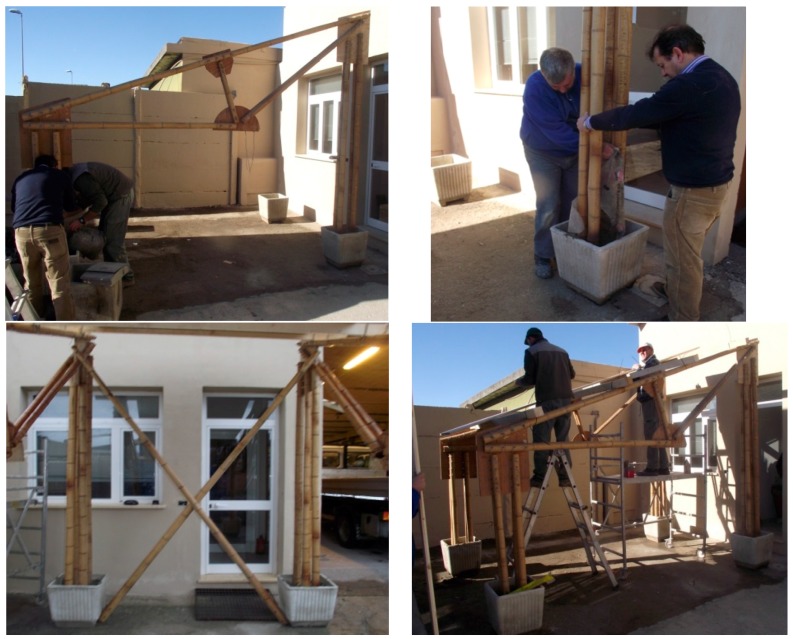
Assembly phases of the 1st full scale specimen.

The assembly phases have been the following (Figure 7):

(a). composition of each beam truss to work site;
(b). connection of each beam truss with two pairs of bamboo columns;
(c). erection of each single bamboo frame (truss/columns), inserting each base column into a precast concrete pot with pressed sand;
(d). execution of bamboo bracing to absorb out-of-plane loads on the frames;
(e). construction of the roof made by wooden purlins (6 cm × 6 cm) and tables (thickness 22 mm).

**Figure 8:**
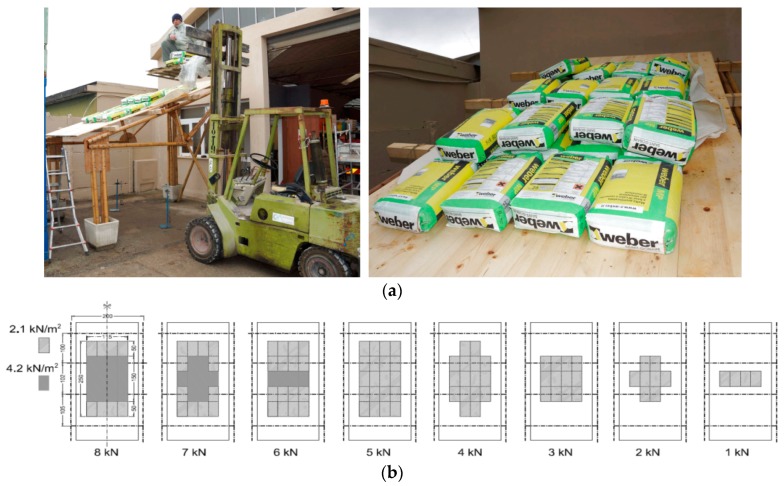
Unit A: application of test loads on the roof (**a**) with loading sequence (**b**).

**Figure 9:**
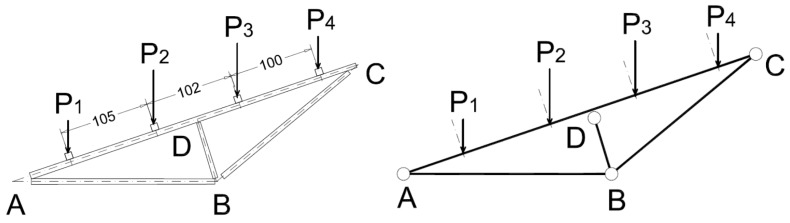
Applied loads on the bamboo rods and internal axial forces.

**Figure 10:**
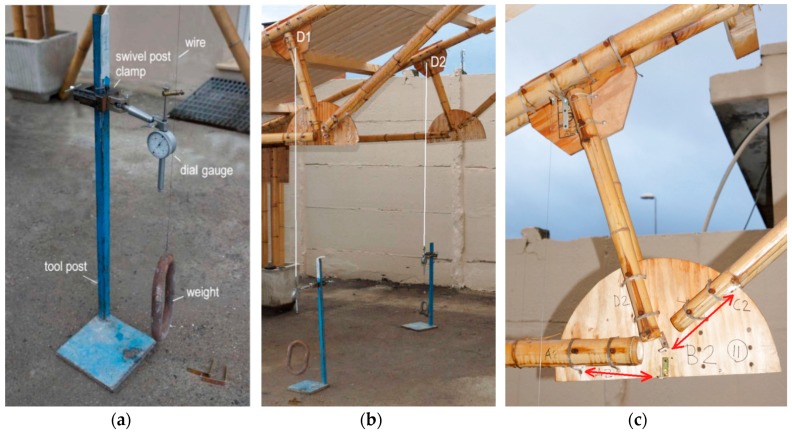
Setup of displacement measurements at node D (**a**,**b**) and node B (**c**).

**Figure 11:**
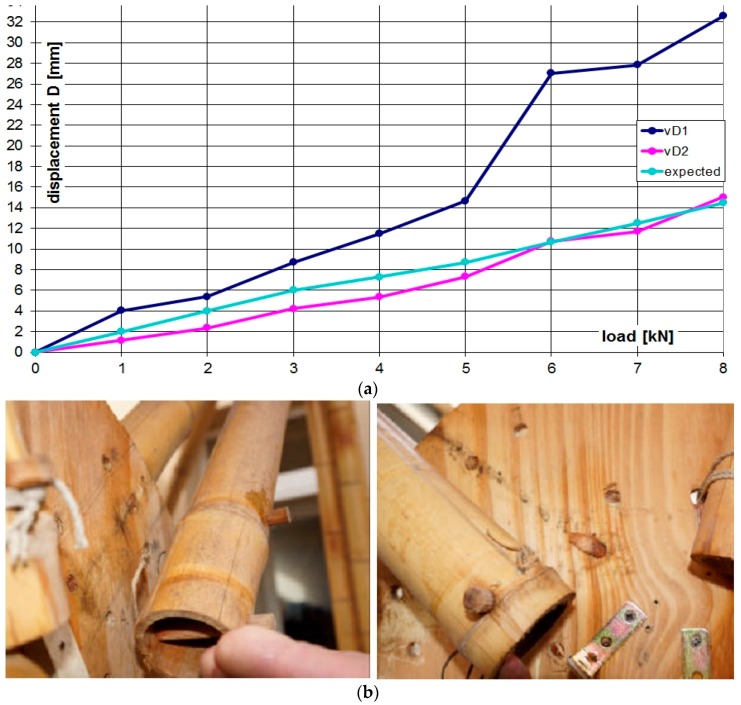
Displacements of node D on both frames and experimental values (**a**); failure of wooden pins (**b**).

**Figure 12:**
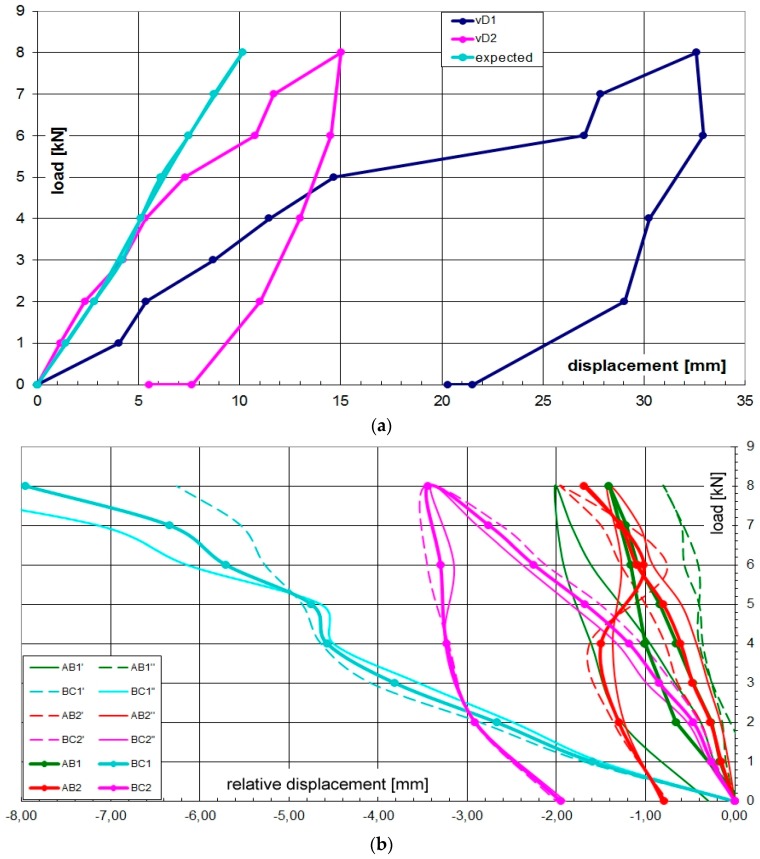
Load-displacement (average values) diagrams of node D (**a**) and node B (**b**).

The static vertical loads were applied throughout a series of cement bags of 25 daN weight, CE certified with a tolerance less than 1%. The loading program was divided into eight steps at 100 daN each with the sequence displayed in Figure 8. The maximum vertical load corresponds to an equivalent uniform pressure of about 1.30 KN/m^2^. The load steps and the axial force in the rods of the truss are shown in Figure 9. The setup of the measurements is conceived to permit easy replacement in a low technology context. During the test (Figure 10) the vertical displacement components of the node D (beam mid-node) were measured by mechanical strain gauges (tolerance 1/100 mm) fixed to the ground, and the relative displacements between the rods AB and BC with respect to the plate in B were measured by removable mechanical strain gauges (tolerance 1/100 mm).The whole testing procedure took place at the Laboratory of Structural Engineering of the University of Pisa. The diagrams of the vertical displacement of node D of both frames (v_D1_, v_D2_) are shown in Figure 11, together with the expected values obtained considering a Young modulus of bamboo *E* = 3000 MPa. Higher values of the displacements were detected in the frame 1 (32 mm) with respect to frame 2 (15 mm). This is due to more significant damages on nodes and imperfections on bamboo canes of frame 1. A visible crack during the 6th load step was observed on the pins at node B of the rod BC. The other nodes did not reveal visible damages. All the relative displacements histories in B are reported in Figure 12.

The maximum value of the testing loads was progressively decreased in four steps (2.0 KN each). The vertical displacements of the node D of both frames are displayed in Figure 11. One hour after completing the unloading phase, the displacement showed a tendency to reduce as a consequence of a visco-elastic component of about 10% of residual deformation. The load-relative displacement diagram of the B-node and the load-displacement diagram of the D-node are shown in Figure 12.

It can be observed that the bamboo structure has been protected from risks of brittle collapses and from failures with complicated repair; this is due to the role of the wooden pins. Also the occurrence of a failure in a node at the 6th stepdid not prevent the structure from taking additional load steps up to the 8th, demonstrating the remarkable inelastic behavior of the entire frame after a local collapse. That is of primary importance to ensure people's safety and also the safety of the related investment.

After the full scale experiment, it was sufficient to renovate the failed wooden pins and to apply a new set of canapé rods in the collapsed node. The repairing activity was performed easily and in a short time (less than one hour). The loading program was then repeated without any inconvenience. It can also be noticed that the tests have been conducted in the absence of horizontal forces. However, the shear and bending moment on the columns, due to transversal forces simulating seismic or wind action, did not induce further stresses on the trussed roofs, if a hinged joint between truss and columns is adopted. Moreover, the increment of axial force on the truss elements, given by horizontal forces on the roof can be easily evaluated by numerical analysis: the lightweight of the roof anyway imply not relevant seismic effects. Further tests can be carried out to experimentally investigate this aspect.

### 4.2. The 2nd Full Scale Model (Unit B)–Assembling Tests

**Figure 13:**
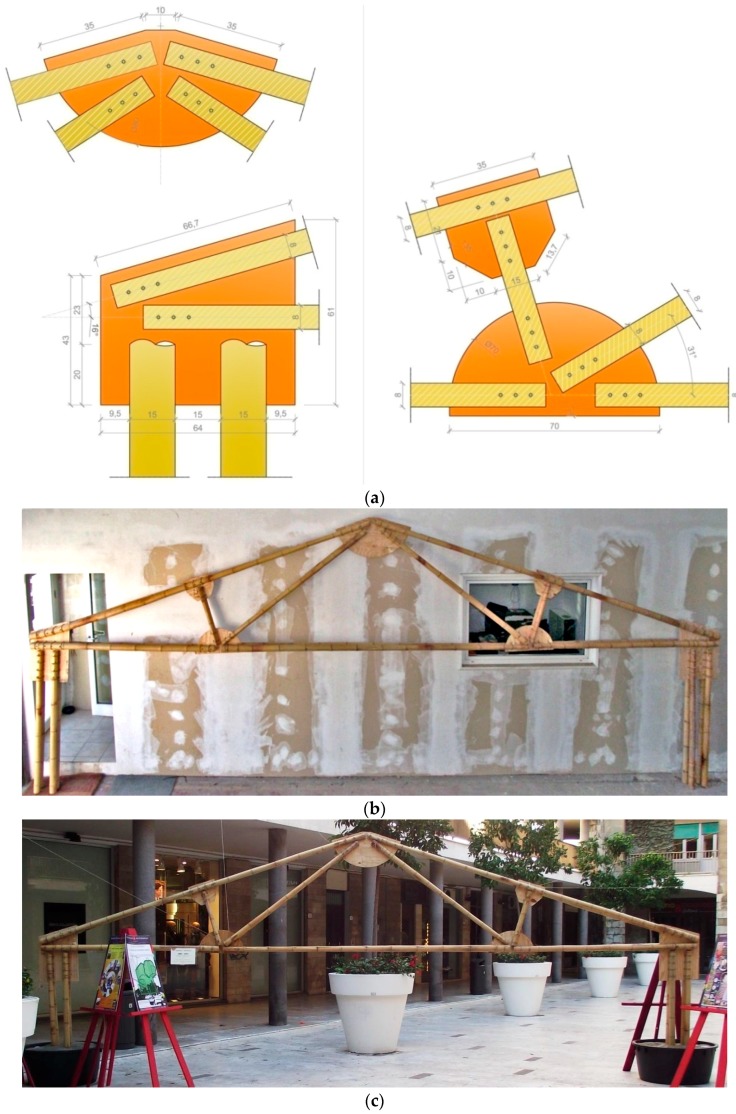
Unit B: node measures in cm (**a**); assembly tests in laboratory (**b**) and in an outside space (**c**).

The third joint type was adopted in another full scale model (Unit B) described in Figure 13. It is based on the classic Polonceau truss scheme to arrange a double deck bamboo frame with a span of about 7.50 m. The specimen was subjected to a service load test and two assembling tests in two different conditions. One test was conducted in laboratory (Figure 13b), the other one in an outside space (Figure 13c), to verify, in both situations, the feasibility of the assembling procedure.

The assembling tests were conducted by unqualified workers, to demonstrate the simplicity of the constructive phases and the ease of the dissemination phase to unskilled personnel. Each Polanceau beam of span L = 7.50 m has a self-weight of about 40 daN, allowing them to be handled without elevator devices. The entire frame was assembled in about 120 min by three volunteers in both conditions, using only a drill, a saw and a hammer.

It is relevant to point out that the mechanical behaviour of several nodes (Figure 13a) is covered by the experiments on single rods: in this case the joint collapse affects only the wooden pins (mechanism n.1 in Section 2) avoiding failure of the other elements. It is also possible to vary the frame span in a proper range using the same node types, particularly the same plywood plates, permitting precast solutions for several values of the main span (for Unit B it has been evaluated a range of span from 6.5 m to 8.5 m).

## 5. Constructive Solutions for One-Storey Buildings

**Figure 14:**
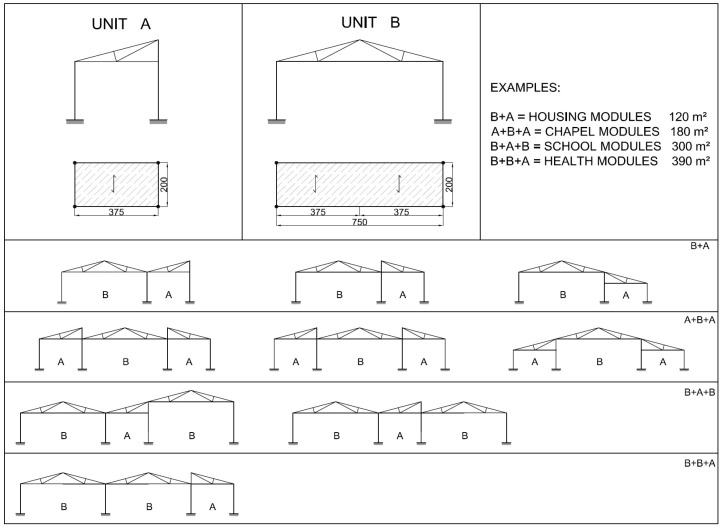
Schemes for unit A and B compositions.

**Figure 15:**
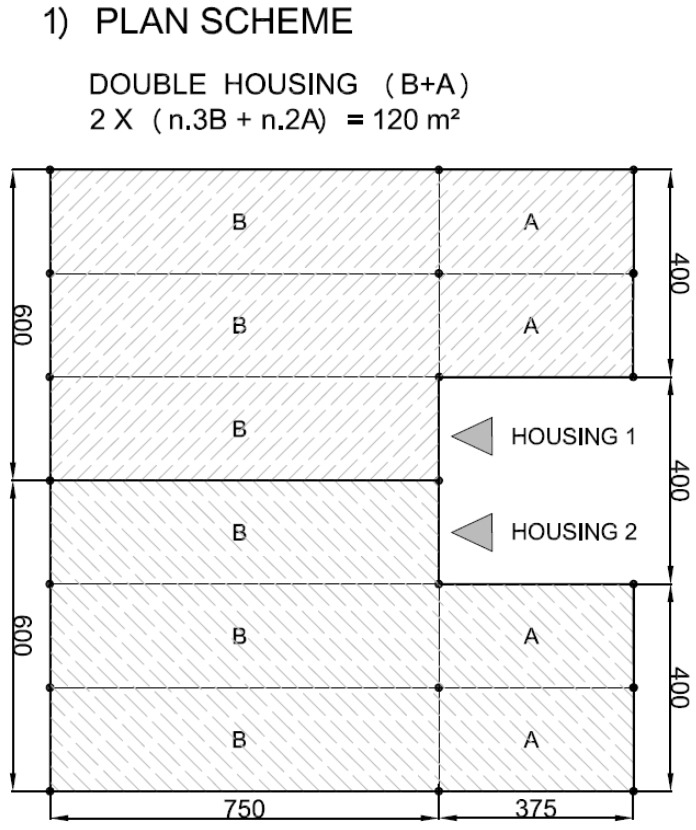
Example of plan view for housing with units A and B.

**Figure 16:**
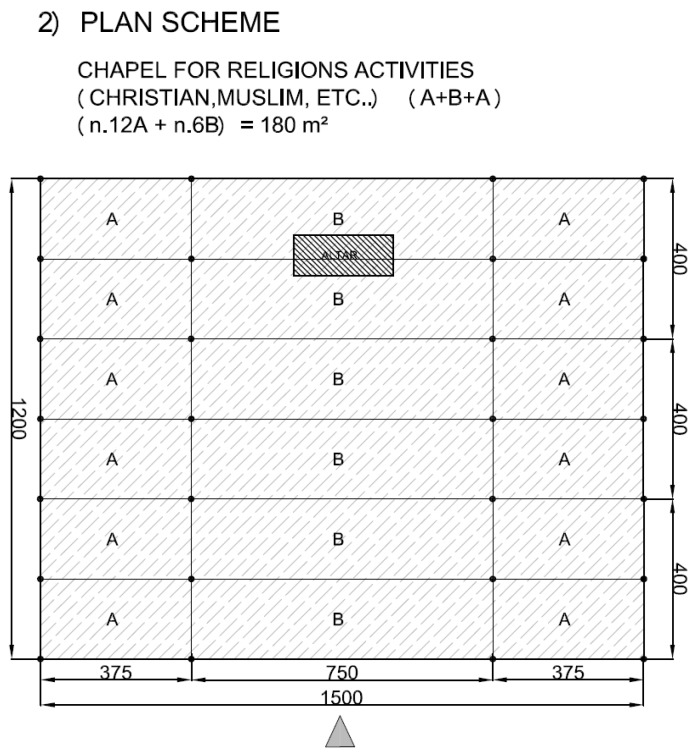
Example of plan view for religious centre with units A and B.

**Figure 17:**
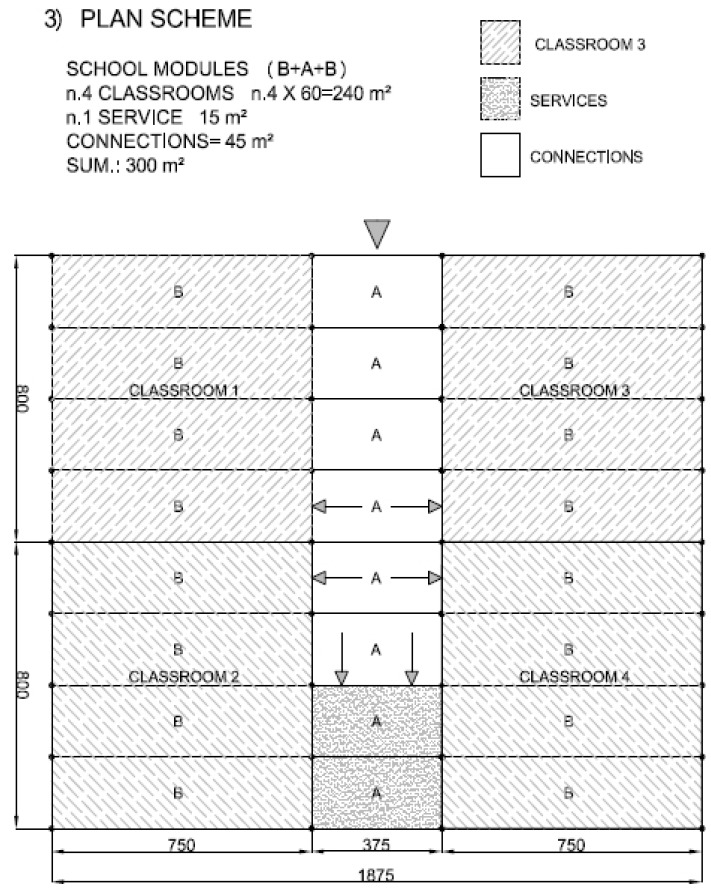
Example of plan view for school building with units A and B.

**Figure 18:**
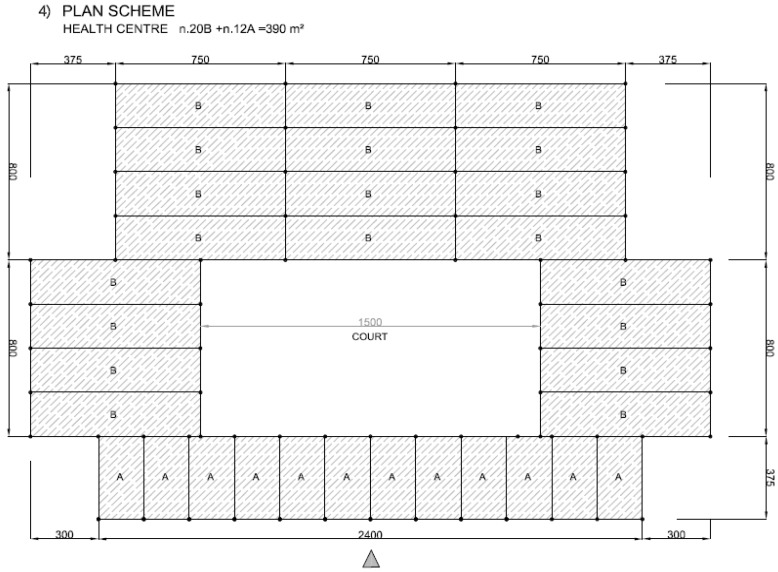
Examples of plan view for health centre with units A and B.

The structural solutions here tested through full scale frames can be easily implemented in different types of buildings, which are common in a large number of states like India, Latin America, East Asia or Sub-Saharan Africa [40,41,42,43], most of them rich in bamboo. A set of modular solutions are here proposed, based on the schemes depicted in Figure 14. The examples in Figure 15, Figure 16, Figure 17 and Figure 18 represent possible arrangements for private or public buildings and can be easily adapted by each designer to fulfill specific needs. The proposed schemes have been organized with the following moduli: A + B (housing); A + B + A (religious centre); B + A + B (school); A + A + B (health centre).

## 6. Discussion

The three proposed types of joint provide different solutions in terms of technology. From the first to the third type, an increasing ductility was detected, together with an increasing simplicity in execution. The increasing ductility has been obtained throughout a progressive weakening of joints. In the first joint the collapse strength was higher but brittle. The use of bolts, internal reinforcement and glue induced a brittle collapse of the bamboo cane. In the second joint the collapse caused wide deformation of the holes in the plywood plate. In the third joint the collapse was associated with the bending moment and shear in wooden pins showing large deformations. This has been made possible by removing the internal reinforcement (due to wooden cylinder in the first joint and bamboo sticks in the second joint) and coupling the bamboo canes to symmetrically connect them to the plywood plates. Tensile stress with a low number of cycles has always characterized all tests. Further experiments should be programmed to explore the mechanical behavior of the joints in presence of high number of cycles.

The third type of joint is easy in execution, in maintenance and in repair. It is also simple to design, taking into account that the diameter and number of the wooden pins can be easily evaluated throughout ISO/DIS 22156 Equations (1) and (2). The concern that weakening the joint (from 1st to 3rd solution) could be dangerous for bamboo constructions disappears, considering that the deformation of the wooden pins permits adaptive displacements of the structure, as shown in the full scale collapse test. Any brittle collapse is thus avoided. Moreover, the weakening of the bamboo canes by holes is common to many solutions presented in technical codes [19] or in literature [28]. It has to be pointed out that a proper protection of the joints from weather and from organic attacks is crucial to ensure the maintenance of the structural performances during time.

In this work the full collapse test has been limited to monotonic vertical loads. Moreover, as convenient strategy in case of on-site tests, the experimental apparatus has been intentionally limited, ensuring sufficient accuracy in the measurements of the displacements (tolerance 1/100 mm) [44].

Further experiments should be conducted to test the bamboo frame under horizontal and cyclic time history loads. Regardless, in this case, due to the structural scheme with hinged joints between the bamboo truss and the columns, the horizontal loads induce bending moments and shear loads only in the columns. To emphasize the dissipating properties, the connections of the columns to the ground can be designed to perform seismic base-dissipation taking into account the “biaxiality effect” induced by the restraints at the base [45].

The constructive technique proposed here is easy to disseminate especially in earthquake prone areas: the significant difference in the loss of life caused by earthquakes in developing countries can be mitigated by spreading construction practices characterized by simplicity, together with the setting up of tutorials and international networks to promote collaboration and information sharing [46]. In this sense, the success of the assembly tests carried out by unskilled operators is an example of the feasibility of the proposed solution. Further assembling tests can be performed in developing countries to verify this aspect.

Finally the set of plan schemes from the presented frames is an attempt to transfer the results to practical applications, offering suggestions for possible destinations. Obviously, the structural aspect is not the only one to investigate: other properties should be analyzed, such as vertical walls type, finishing, covering, *etc*.

## 7. Final Comments and Conclusions

In this work three different types of bamboo joints are proposed. From the first to the third type, an increasing ductility was detected, together with an improvement of simplicity in execution. The third type of joint provides easy construction and simple repair procedures, a reduction of the level of technology providing environmental friendly materials, and satisfactory structural performances. The strategy of the “weak pins” leads joints to ductile collapses protecting bamboo from brittle failures and the nodes from complicated repairs. The cyclic tests show the hysteretic behavior of the structure.

Two different full scale one-storey trusses show the structural efficiency and the feasibility of the system for construction and repair. The proposed bamboo joints can also be applied for a wide range of span trusses, allowing for a precast structural system. The ease of execution and assembly is a relevant feature of the proposed frames, permitting dissemination and maintenance in low-technology areas and promoting self-construction or self-enterprise for local communities, together with the subsequent maintenance activity.

The application of the proposed technique is not exclusive for developing countries. It can be also adopted in all the areas with environmental and technological features for the use of bamboo constructions, in view of its eco-friendly characteristics. Moreover the structural system involves only natural materials (bamboo canes, plywood plate, wooden pins, canapé ropes) with strong reduction of environmental impact in those areas where the natural materials are available.

Further investigations should be conducted to protect the façade walls (here not considered) from the risk of rocking falls from the movements of the roof, as in [47,48], due to earthquakes. It will be carried out on additional structural schemes and corresponding experiments. Furthermore it will evaluate the influence of cyclic loadings on the whole structure and on the single joints of the bracing system. Experiments with a large number of cyclic steps and in the presence of inversion of the axial force (tension-compression), related to wind and seismic actions, can help to investigate this aspect.

## Figures and Tables

**Table:** 

Total Load	P1	P2	P3	P4	N_AD_	N_DC_	N_AB_	N_BC_	N_BD_
8 kN	17	190	185	15	−497	−437	490	508	−338
7 kN	15	162	160	14	−444	−394	421	437	−290
6 kN	15	139	135	14	−343	−323	361	374	−250
5 kN	19	110	106	18	−292	−260	292	303	−202
4 kN	8	95	93	7	−246	−218	245	254	−170
3 kN	−3	79	78	−3	−194	−173	193	200	−133
2 kN	−3	53	53	−3	−131	−117	130	135	−90
1 kN	−3	28	28	−3	−69	−60	67	70	−46
